# A group‐based hands‐on seminar for teaching failure mode and effects analysis in CyberKnife stereotactic radiotherapy

**DOI:** 10.1002/acm2.70789

**Published:** 2026-09-10

**Authors:** Hiroyuki Okamoto, Shie Nishioka, Takeshi Kamomae, Kohei Okawa, Souichi Kikuchi, Junji Suzuki

**Affiliations:** ^1^ Section of Radiation Safety and Quality Assurance National Cancer Center Hospital Tsukiji, Chuo‐ku Tokyo Japan; ^2^ Department of Radiation Oncology Japanese Red Cross Kyoto Daini Hospital Kamigyo‐ku, Kyoto Kyoto Japan; ^3^ Medical Branch, Radioisotope Research Center Nagoya University Showa‐ku, Nagoya Aichi Japan; ^4^ Department of Radiology Nagoya University Graduate School of Medicine Showa‐ku, Nagoya Aichi Japan; ^5^ Department of Radiotherapy Quality Management Shinryoku Neurosurgical Clinic, Yokohama CyberKnife Center Yokohama Kanagawa Japan; ^6^ Faculty of Health Sciences Butsuryo College of Osaka Sakai Osaka Japan; ^7^ Division of Health Sciences, Graduate School of Medical Sciences Kanazawa University Kanazawa Ishikawa Japan; ^8^ Radiotherapy Quality Management Group TOYOTA Memorial Hospital Toyota Aichi Japan

**Keywords:** CyberKnife, failure mode and effects analysis, quality management, risk analysis, stereotactic radiotherapy

## Abstract

**Background:**

Risk analysis is becoming increasingly important in advanced radiotherapy, as treatment workflows grow more complex and require multidisciplinary expertise. Although failure mode and effects analysis (FMEA) has been recommended for risk‐based quality management, opportunities for users to practically learn and apply FMEA within CyberKnife® workflows remain limited.

**Purpose:**

To evaluate the feasibility and educational value of a group‐based, hands‐on seminar for teaching FMEA methodology in CyberKnife® stereotactic radiotherapy, using four representative treatment sites as practical examples.

**Methods:**

A half‐day seminar was conducted for eight radiation technologists and six medical physicists from CyberKnife® institutions, supported by four facilitators. Following a didactic lecture on risk analysis, participants were divided into four site‐specific groups (intracranial, lung, spine, and prostate). Each group utilized institution‐prepared process maps and a shared Excel‐based worksheet to identify potential failure modes, score them using a five‐point FMEA scale for occurrence, severity, and detectability, and discuss possible countermeasures. Risk priority numbers (RPNs) were calculated to rank the identified failure modes. A postseminar questionnaire was administered to assess participants’ prior experience, level of understanding of risk analysis, and willingness to implement it in clinical practice.

**Results:**

The seminar generated site‐specific failure mode lists as educational outputs. The highest‐ranked risks were contouring errors for intracranial lesions (RPN 48), irregular respiration for lung lesions (RPN 64), pain‐induced patient motion for spinal lesions (RPN 36), and target delineation errors for prostate lesions (RPN 80). Other major risks included inappropriate selection of immobilization devices, prescription and fractionation errors, tracking‐related issues, image registration inaccuracies, and incorrect patient‐specific QA evaluation criteria. Thirteen participants completed the questionnaire. Most participants had limited prior experience with risk analysis; however, 85% reported a good level of understanding after the seminar, and 92% expressed willingness to implement risk analysis at their institutions.

**Conclusions:**

This group‐based seminar provided participants with practical experience in applying FMEA to CyberKnife treatment workflows. The questionnaire responses suggested that most participants gained a better understanding of risk analysis and were interested in applying it at their own institutions. The failure mode lists generated during the seminar were not intended to represent a complete FMEA for any participating institution but may provide practical examples for initiating multidisciplinary risk analysis based on local workflows and clinical practices.

## 1 INTRODUCTION

Risk analysis, a systematic approach for identifying, evaluating, and mitigating potential hazards, has long been recognized as essential in both industrial and medical fields. In radiotherapy, its importance has further increased owing to the growing complexity of treatment modalities and the expanding multidisciplinary expertise required for the safe delivery of advanced therapies. The American Association of Physicists in Medicine (AAPM) Task Group 100 (TG‐100) report established a foundation for the application of risk analysis to quality management in radiation therapy.^1^ Conventional quality assurance (QA) guidelines, such as AAPM TG‐142,^2^, ^3^ primarily focus on periodic quality control of treatment machines. In the context of robotic radiosurgery, AAPM TG‐135 and its updated report, TG‐135.B, provide technology‐specific QA recommendations for systems such as CyberKnife®.^4^, ^5^ In contrast to these technical QA guidelines, TG‐100 emphasizes a comprehensive, process‐based approach. This framework enables the systematic identification of vulnerabilities within clinical workflows and supports the development of specific mitigation strategies tailored to those vulnerabilities. In this context, TG‐100 is not intended to replace TG‐142; rather, it complements it by addressing challenges associated with the clinical implementation of new and complex treatment technologies.

CyberKnife® is a robotic radiosurgery system characterized by high precision, non‐isocentric beam delivery, and real‐time image guidance, and it represents a prominent example of both the challenges and opportunities in modern radiotherapy.^6^ As CyberKnife® technology continues to evolve, the complexity of its clinical application has also increased. Accordingly, a proactive risk management strategy is required—one that is grounded in established technical QA recommendations, including AAPM TG‐142^2^, ^3^ and the CyberKnife®‐specific guidance provided by AAPM TG‐135,^4^, ^5^ while also incorporating the process‐oriented perspective advocated by TG‐100.^1^ Through such an integrated approach, institutions can establish robust safety frameworks capable of addressing the unique demands of advanced treatment delivery systems.

In response to these challenges, we organized a hands‐on seminar specifically tailored for CyberKnife® users. To our knowledge, this seminar represents one of the first practical training initiatives for CyberKnife® practice to explicitly incorporate TG‐100‐based risk analysis. It offered participants the opportunity to acquire a solid understanding of the fundamental principles of AAPM TG‐100 and to apply risk analysis methodologies to the workflow specific to CyberKnife® treatments. By combining theoretical instruction with structured practical exercises, the seminar was designed to help participants systematically identify potential failure modes and discuss appropriate mitigation strategies for clinical practice.

The value of this initiative lies in its educational approach, which bridges established technical QA guidelines, including TG‐142^2^, ^3^ and the CyberKnife®‐specific recommendations of TG‐135,^4^, ^5^ with the process‐oriented framework of TG‐100.^1^ Furthermore, a postseminar survey was conducted to gather detailed feedback on the seminar's effectiveness in enhancing safety culture and improving readiness for local implementation.

The primary objective of this study was to evaluate the feasibility and educational value of a group‐based hands‐on seminar for teaching FMEA methodology in the context of CyberKnife® stereotactic radiotherapy. The FMEA‐derived failure mode lists were regarded as seminar outputs intended to support education and discussion, rather than comprehensive institutional FMEA results for direct implementation in clinical practice.

## 2 MATERIALS AND METHODS

Eight radiation technologists and six medical physicists from institutions equipped with CyberKnife® systems participated in the seminar. In addition, four facilitators from the organizing team were involved. The seminar was conducted as a half‐day program and comprised two main components: A didactic lecture session and a hands‐on practical session. In the didactic session, a comprehensive overview of risk analysis was delivered using failure mode and effects analysis (FMEA)^1^ as the primary framework. This lecture lasted approximately 50 min and introduced the theoretical foundations of risk analysis, with particular emphasis on its application to advanced radiotherapy practice.

**FIGURE 1:**
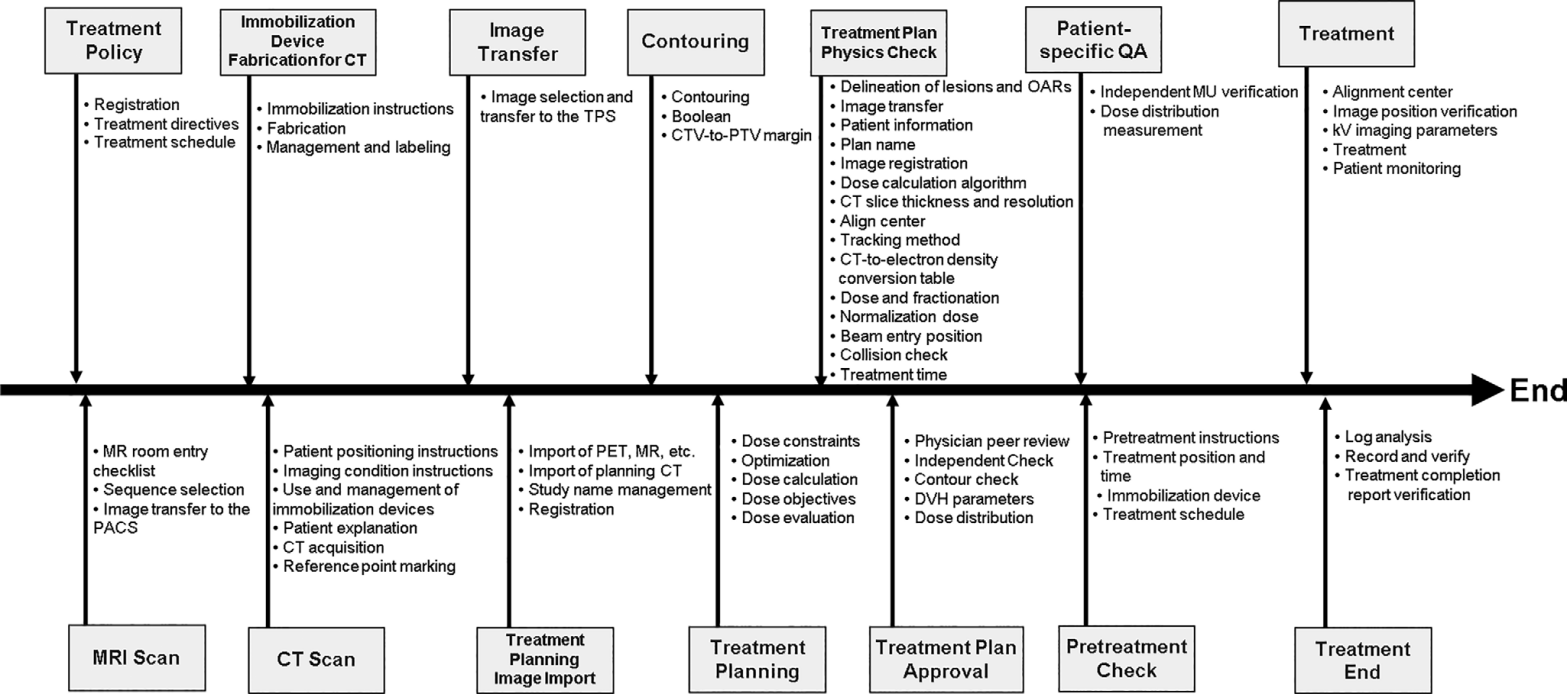
Example of a process map for intracranial lesion treatment using CyberKnife®.

The hands‐on session, lasting approximately 180 min, was designed to provide participants with structured practical experience in risk analysis. Participants were divided into four groups according to treatment sites using CyberKnife®: Intracranial, lung, spine, and prostate. Before the seminar, we surveyed each institution to determine the treatment sites they performed in clinical practice. Based on this information, participants were assigned to groups so that the number of members in each group was balanced. Each group was assigned a facilitator to support the process and ensure the smooth progression of the risk analysis activities. Prior to the seminar, each participating institution prepared a process map outlining its clinical workflow. Figure 1 shows an example of a process map for intracranial lesion treatment using CyberKnife®. These process maps served as the basis for group discussions, during which each institution presented its treatment procedures, thereby promoting interinstitutional communication and exchange of best practices. In addition, each facilitator shared a Microsoft Excel® spreadsheet via a monitor prepared for each group to document failure modes and FMEA scores (Figure S1). The facilitators also recorded the identified failure modes, corresponding scores, and proposed countermeasures from each group, including desired functions for future implementation. The hands‐on exercise proceeded as follows. (1) Identification of failure modes: Within a limited time, each group conducted brainstorming using a preconfigured Excel spreadsheet displayed on a monitor and listed potential failure modes (Figure S1). Groups were instructed not only to report failure modes experienced at their own institutions but also to share those observed at other institutions and to identify potential failure modes that had not yet occurred but could arise in the future. (2) Scoring: Each failure mode was evaluated using a five‐point scale across three dimensions: Frequency of occurrence (*O*), severity of impact (*S*), and detectability (*D*). To address challenges associated with applying a uniform scoring scale, baseline failure modes were first selected and scored, and these baseline values were subsequently used as reference points for evaluating other failure modes. Although the 10‐point scale proposed in TG‐100 was initially considered, a five‐point scale was ultimately adopted to avoid excessive discussion time and to maintain efficiency during the hands‐on exercise. The scoring criteria are shown in Table 1. (3) Risk prioritization: The identified failure modes were then ranked in descending order according to their risk priority number (RPN), and each group discussed appropriate countermeasures for the highest‐risk items. (4) Presentation: Finally, each group presented the failure modes considered to be of high risk, along with recommended mitigation strategies. At the conclusion of the seminar, a questionnaire survey was administered to evaluate its overall effectiveness. The survey assessed participants’ understanding of risk analysis principles and explored potential barriers to implementing such analyses in their clinical practice.

**TABLE 1:** Scoring criteria for the five‐point FMEA system used in this study. FMEA: Failure mode and effects analysis.

Score	Occurrence (*O*)	Severity (*S*)	Detectability (*D*)
1	Rarely occurs	No dosimetric impact	Definitely detectable
2	Occasionally occurs	Negligible dose error	Easy to detect
3	Occurs from time to time	Minor dose error	Moderately difficult to detect
4	Occurs repeatedly	Major dose error	Difficult to detect
5	Occurs inevitably	Severe dose error	Very difficult to detect

## 3 RESULTS

Failure modes were identified separately by each site‐specific group, followed by a five‐point FMEA scoring process. The representative high‐priority failure modes for each treatment site are summarized in Table 2, including their corresponding RPNs and representative countermeasures. The complete failure mode lists generated during the seminar, including all scores and countermeasures, are provided in Tables S1–S4. Owing to limited discussion time, the analysis primarily captured common failure modes encountered in routine clinical practice. A summary of the site‐specific failure modes is provided below. When descriptions of countermeasures in the submitted Excel worksheets were unclear or insufficient, the coauthors reviewed the entries and appropriately revised or supplemented the proposed mitigation strategies after the seminar. In addition, a direct comparison of RPN values across disease sites should be avoided, as FMEA was conducted independently by different site‐specific groups.

**TABLE 2:** Representative high‐priority failure modes identified for each treatment site during the seminar.

Treatment site	Failure Mode	*RPN*	Countermeasures^*^
Intracranial	Contour inaccuracy	48	Physician peer review and checklist (small‐volume contour and margin)
Intracranial	Incorrect immobilization device type	45	Immobilization device organization and fixation protocol
Intracranial	Incorrect prescription or fractionation instructions	40	Institutional protocol confirmation and information sharing for irregular cases
Intracranial	Diagnostic imaging error	20	Physician peer review
Intracranial	Incorrect identification of the target image location	20	Physician peer review, physics review, and composite evaluation with previous plans, coordination with other departments
Lung	Irregular patient breathing	64	Respiratory management through coaching and visual feedback
Lung	Inadequate consideration of interference between the arm and the machine during the immobilization setup	60	Standardization of treatment positioning, collision check with the machine, and selection of the arms‐down position to avoid collision
Lung	Contact between the machine and the patient during treatment	60	Establishment of an immobilization protocol, standardization of treatment positioning, checking of immobilization device size, and selection of the arms‐down position to avoid collision
Lung	Inappropriate fiducial marker position	48	Information sharing with other departments and training for marker implantation
Lung	Fiducial marker migration	48	Training for marker implantation and check for marker migration (excluding symmetrical markers)
Spine	Patient motion caused by pain	36	Enhanced patient assessment, confirmation at simulation, pain control with medication in collaboration with physicians, and motion monitoring by radiation technologists during treatment
Spine	Change in immobilization device shape during treatment (e.g., air leakage or deformation)	27	Pre‐use check
Spine	Misregistration between MRI and CT	24	Physician peer review, physics review, and checklist (image registration)
Spine	Insufficient calculation range	24	Checklist (calculation range) and multidisciplinary check
Spine	One‐vertebra misalignment during tracking	24	Physician peer review, physics review, double‐check, education on image interpretation skills, and standardization of the vertebral body confirmation procedure in a manual
Prostate	GTV delineation error	80	Physician peer review
Prostate	Incorrect patient‐specific QA evaluation criteria (potentially persistent error)	75	Pre‐use verification during commissioning, manual development, education/training, physician peer review, and physics review
Prostate	Non‐optimal fiducial marker placement	60	Operator training and information sharing with other departments
Prostate	Inadequate spacer placement	60	Operator training and information sharing with other departments
Prostate	Incorrect CT image selection for retreatment planning	40	Physician peer review, physics review, and checklist (CT image selection)

* The countermeasures were revised by the authors based on the outcomes of group discussions. RPN values should not be directly compared across disease sites because each group conducted FMEA independently.

Abbreviations: D, detectability; FMEA, failure mode and effects analysis; O, occurrence; S, severity.

### 3.1 Intracranial treatments

In the risk analysis of CyberKnife® stereotactic radiotherapy (SRT) for intracranial lesions, failure modes associated with contouring accuracy, immobilization device selection, and prescription settings exhibited relatively high RPNs. The highest RPN was observed for dose distribution errors resulting from contour inaccuracy (“small volume” in the contour), with an RPN of 48 (occurrence, 3; severity, 4; detection difficulty, 4). As this item directly affects target volume delineation, it was identified as a critical risk in intracranial stereotactic irradiation. The second highest RPN was associated with dose errors due to the incorrect selection of immobilization device type, with an RPN of 45 (occurrence, 3; severity, 3; detection difficulty, 5). This failure mode was characterized by particularly high detection difficulty, indicating that inappropriate immobilization device selection may adversely affect both treatment planning and delivery while remaining difficult to identify. In addition, incorrect prescription and fractionation settings yielded an RPN of 40 and demonstrated the highest severity score (severity, 5). Because fractionation parameters directly influence treatment efficacy and normal tissue toxicity, this was recognized as a major clinical risk in intracranial stereotactic irradiation. Several failure modes, including diagnostic imaging errors, target misidentification, margin setting errors, and image fusion misalignment, had an RPN of 20. These items were consistently associated with high severity and were linked to target definition or positional accuracy, thereby constituting a significant risk category in intracranial stereotactic irradiation. In particular, image‐based target delineation and margin determination were identified as factors directly affecting the accuracy of dose distribution. Failure modes with an RPN of 18 included incorrect labeling of immobilization devices and communication errors regarding immobilization methods, both of which were related to patient fixation and setup. Automatic contouring errors showed an RPN of 12 and were identified as contributors to reduced contour accuracy. Although several items, such as incorrect collimator placement, inappropriate slice thickness selection, communication errors regarding treatment protocols, incorrect prescription settings, and patient misidentification, exhibited relatively low RPN values, some of these still had high severity scores and could therefore result in serious clinical consequences if they occurred. Overall, for CyberKnife® SRT of intracranial lesions, the primary risk domains were identified as contouring and image‐based target delineation, immobilization device selection, and prescription/fractionation settings.

### 3.2 Lung treatments

In the risk analysis of CyberKnife® SRT for lung lesions, failure modes associated with respiratory motion, tracking accuracy, and patient–treatment unit interference demonstrated high RPNs. The highest RPN was observed for dose distribution deviations resulting from irregular respiration, with an RPN of 64 (occurrence, 4; severity, 4; detection difficulty, 4). This failure mode directly reflects the impact of respiratory motion and was identified as a major risk in lung stereotactic irradiation. The next highest RPNs were observed for contact between the patient and the treatment unit due to arm movement and for treatment interruption caused by patient–machine contact, both with an RPN of 60. Although these failure modes showed moderate severity, they were characterized by high detection difficulty and were considered to be associated with intratreatment positional changes as well as limitations in the robotic arm's motion range. Fiducial marker displacement also demonstrated an RPN of 48, contributing to both the reduced tumor dose and excessive dose to organs at risk as well as positional deviations during irradiation. These failure modes were directly linked to tracking accuracy and were recognized as important risks affecting both dose delivery and spatial accuracy. Furthermore, changes in respiratory pattern, alterations in the correlation model, and patient movement were each associated with an RPN of 32, all of which contributed to positional deviations during treatment. Incorrect fraction numbers yielded an RPN of 30 and the maximum severity score. In contrast, failure modes such as communication errors regarding immobilization devices, incorrect selection of tracking markers, and image fusion errors exhibited relatively low RPN values. However, these items still had high severity scores and therefore require careful attention due to their potential clinical impact if they occur. Overall, in CyberKnife® SRT for lung lesions, the primary risk domains were identified as respiratory motion management and tracking accuracy, with additional risks related to intratreatment positional changes and patient–machine interference.

### 3.3 Spinal treatments

In the risk analysis of CyberKnife® SRT for spinal tumors, failure modes associated with patient movement, immobilization accuracy, image fusion, and interference with the robotic arm demonstrated high RPNs. The highest RPN was observed for irradiation positional errors caused by patient movement due to pain, with an RPN of 36 (occurrence, 3; severity, 3; detection difficulty, 4). This failure mode reflects a characteristic risk in spinal cases, where pain may increase the likelihood of patient movement during treatment. The second highest RPN was associated with replanning due to changes in the shape of the immobilization device during treatment, with an RPN of 27. This failure mode suggested that deformation of the immobilization device or air ingress could reduce positional reproducibility and was identified as a risk affecting both treatment accuracy and workflow efficiency. Failure modes with an RPN of 24 included magnetic resonance imaging–computed tomography (MRI–CT) registration errors, inappropriate calculation range settings, vertebral misidentification during tracking, and interference between the patient and the robotic system. These items represented a major group of risks related to image accuracy, dose evaluation, positional identification, and mechanical safety. In particular, vertebral misidentification was considered a critical spine‐specific risk, as it could result in irradiation positional errors on the order of several centimeters. Additional risks included incorrect positional judgment during CT acquisition and mismatch in immobilization device sizing. Furthermore, failure to correct imaging artifacts, incorrect calculation resolution settings, and positional shifts in the roll direction showed moderate RPN values; however, all were related to precision management and treatment accuracy. In contrast, incorrect physician input of irradiation instructions and beam‐setting errors exhibited relatively low RPN values. Nevertheless, these items had high severity scores and could therefore lead to serious clinical consequences if they occurred. Overall, for CyberKnife® SRT of spinal tumors, the principal risk domains were identified as pain‐related patient motion, image fusion accuracy, accuracy of bony structure tracking, and interference with the robotic system.

### 3.4 Prostate treatments

In the risk analysis of CyberKnife® SRT for prostate cancer, failure modes associated with target volume delineation, treatment planning, QA, and image data handling demonstrated high RPNs. The highest RPN was observed for significant dosimetric impact resulting from incorrect gross tumor volume (GTV) delineation, with an RPN of 80 (occurrence, 4; severity, 5; detection difficulty, 4). As this failure mode directly affects target definition, it was identified as the most critical risk in prostate stereotactic irradiation. The second highest RPN was associated with the incorrect setting of patient‐specific QA evaluation criteria, with an RPN of 75 (occurrence, 3; severity, 5; detection difficulty, 5). Given its maximum severity and detection difficulty scores, this failure mode was considered a major risk, particularly because inappropriate evaluation criteria may persist undetected and be applied over time. Failure modes with an RPN of 60 included positional deviations due to non‐optimal gold marker placement and dose impact resulting from insufficient rectal spacer placement. These procedure‐specific risks in prostate stereotactic irradiation may affect tracking accuracy and rectal dose sparing. Failure modes with an RPN of 40 included CT image misidentification during replanning, communication errors in treatment planning instructions, and incorrect CT image import. Although these events had relatively low occurrence frequencies, all were associated with a severity score of 5 and could result in major dosimetric errors if they occurred. In addition, incorrect CT slice thickness settings (RPN = 36) and insufficient CT scan ranges (RPN = 32) were identified, indicating that inappropriate image acquisition parameters may adversely affect dose evaluation and positional accuracy. Incorrect pre‐treatment patient preparation instructions, including instructions for bladder filling and rectal emptying, had an RPN of 30 and were considered to potentially affect positional reproducibility and increase the dose to the rectum or bladder. Other failure modes, including incorrect CT selection during QA plan creation (mainly related to QA failure or treatment delay), misalignment of the reference isocenter (related to treatment planning geometry), insufficient MRI image quality, air leakage from the immobilization device, and omission of MRI sequence acquisition, showed relatively low RPN values. However, these items were still identified as factors that could affect image quality and alignment accuracy. Overall, in CyberKnife® SRT for prostate cancer, the highest‐risk domains were associated with planning and evaluation processes, particularly GTV delineation and patient‐specific QA evaluation criteria. In addition, procedural factors involving fiducial marker and rectal spacer use, as well as errors in image acquisition and data handling, were identified as major sources of risk.

**FIGURE 2:**
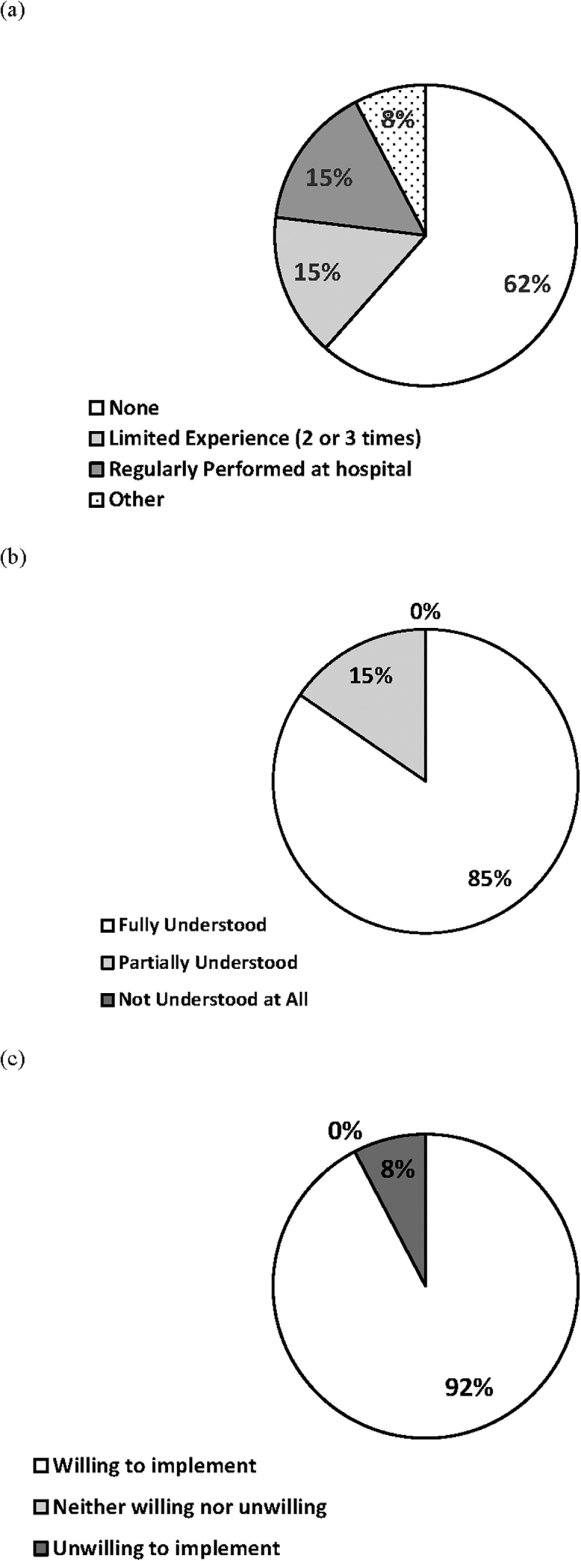
Postseminar questionnaire results from 13 participants: (a) prior experience with risk analysis, (b) understanding of risk analysis after the seminar, and (c) willingness to implement risk analysis in future clinical practice.

Figure 2 summarizes the questionnaire responses from the 13 participants who completed the postseminar survey. With regard to prior experience in risk analysis, most respondents reported limited exposure: 62% (8/13) indicated no prior experience, 15% (2/13) had encountered risk analysis only two to three times in seminars or similar educational settings, 15% (2/13) reported that it was routinely performed at their institution, and 8% (1/13) selected “other” (Figure 2a). These results indicate that the majority of participants had minimal practical experience with risk analysis before attending the seminar. Regarding postseminar understanding of risk analysis, 85% (11/13) of respondents reported a good level of understanding of the content, whereas 15% (2/13) reported partial understanding; no respondents indicated a lack of understanding (Figure 2b). These findings suggest that, despite limited prior exposure among participants, the seminar effectively enhanced their understanding of the purpose and methodology of risk analysis. When asked about their willingness to implement risk analysis in future clinical practice, 92% (12/13) responded affirmatively, whereas 8% (1/13) were unwilling to implement it; no respondents indicated a neutral response (Figure 2c). In the free‐text responses regarding potential barriers to implementation, participants mainly identified the need to secure time for multidisciplinary discussion, obtain cooperation from physicians and other professional groups, and establish institutional leadership and support for risk analysis activities. Overall, these results suggest that the seminar not only improved participants’ understanding of risk analysis but also influenced their intention to incorporate it into future clinical practice.

## 4 DISCUSSION

Previous CyberKnife®‐specific studies on risk analysis have reported FMEA applications for CyberKnife® stereotactic radiosurgery (SRS) and stereotactic body radiotherapy (SBRT).^7^, ^8^ These studies have provided important information on high‐risk steps in CyberKnife® workflows. In contrast, the primary contribution of the present study is not the identification of previously unreported CyberKnife®‐specific failure modes, but the implementation of a group‐based, hands‐on educational seminar for teaching TG‐100‐based FMEA methodology to CyberKnife® users.

In this seminar, participants were organized into disease‐site‐specific groups and used process maps and structured worksheets to identify failure modes, score risks, and discuss potential countermeasures. The failure mode lists generated during the seminar should therefore be interpreted as educational outputs that supported discussion and learning, rather than as comprehensive institutional FMEA results. At the conclusion of the seminar, each group presented its key findings and proposed countermeasures, which allowed participants to learn not only from their assigned treatment site but also from the risk analysis processes applied to other treatment sites.

The present study generated site‐dependent but clinically coherent risk profiles. In intracranial treatments, key concerns included contouring accuracy, immobilization device selection, and prescription and fractionation parameters. In lung treatments, major issues involved respiratory motion irregularity, tracking accuracy, and potential collisions between the patient and the robotic system. In spine treatments, important risks were associated with pain‐induced motion, image registration accuracy, vertebral level identification, and robotic system interference. In prostate treatments, primary concerns included target delineation, patient‐specific QA evaluation criteria, fiducial‐related procedures, spacer placement, and image data management. Collectively, these findings indicate that CyberKnife®‐related risks are influenced not only by machine‐specific characteristics but also by treatment sites, tracking methodologies, imaging workflows, institutional treatment policies, and local QA practices.

The multi‐institutional format allowed participants to share experiences from different clinical environments, including variations in treatment protocols, system versions, and QA practices. This exchange may have helped participants recognize workflow vulnerabilities that can be difficult to identify through discussion within a single institution. However, because the seminar was time‐limited and educational in nature, the resulting failure mode lists should not be regarded as comprehensive or universally applicable. Each institution should conduct its own multidisciplinary FMEA based on local workflows, clinical practice, and available resources.

The questionnaire results further support the educational value of this initiative. Although most participants had limited prior experience with risk analysis, the postseminar survey demonstrated that 85% reported a good level of understanding of the concept after the seminar, while 92% indicated their intention to implement risk analysis at their respective institutions in the future. These findings are particularly noteworthy, as they suggest that the seminar did not merely provide a list of failure modes but instead enhanced participants’ practical understanding of FMEA and increased their motivation to apply it within their clinical environments. In many institutions, the importance of risk analysis is acknowledged in principle; however, its implementation remains limited because staff may be uncertain about how to begin, may lack confidence, or may not have opportunities for multidisciplinary discussion. The combined use of didactic instruction and site‐specific, hands‐on group work in this study appears well suited to addressing these barriers. From this perspective, such an approach may represent an effective entry point for the broader implementation of TG‐100‐based risk management in CyberKnife® practice.^1^ Furthermore, existing literature on incident learning in radiation oncology indicates that safety culture is strengthened when near misses and incidents are systematically analyzed, fed back to users, and shared across a wider professional community.^9^

The value of this seminar format lies primarily in hands‐on education in the FMEA process, rather than in the repeated identification of novel failure modes. Although the common failure modes observed across users support the educational validity of the seminar outputs, these outputs should not replace a comprehensive, multidisciplinary, institution‐specific FMEA. Broader implementation of this seminar format may therefore help participants become familiar with TG‐100‐based risk analysis and facilitate subsequent local FMEA implementation.

The 10‐point scoring system proposed in AAPM TG‐100 has been widely adopted in radiation oncology FMEA because it allows finer discrimination among failure modes.^9^ However, simplified or modified scoring systems have also been reported and may offer practical advantages by facilitating score assignment and reducing the evaluative burden.^10^, ^11^, ^12^ In the present seminar, a 5‐point scale was adopted because of limited discussion time and the need for efficient scoring within a multigroup workshop setting. Although this approach reduces score granularity compared with a 10‐point scale, it was sufficient to characterize the relative risk profiles of each treatment site. Therefore, in view of the practical and educational objectives of this seminar, the 5‐point scoring system was considered appropriate.

Several limitations of this study should be acknowledged. First, as this was a one‐day workshop, the time available for discussion was necessarily constrained. Consequently, the identified failure modes were likely influenced by participants’ direct clinical experience and may have been weighted toward more common and readily recognizable events. While a key strength of FMEA is its ability to capture not only known failures but also rare, unexpected, or low‐frequency events with potentially severe consequences, the present format may have limited the depth of exploration in this regard. Second, the participants were limited to medical physicists and radiation technologists. The absence of physicians, nurses, and other clinical personnel may have biased the analysis toward technical aspects of treatment planning, quality assurance, and treatment delivery, rather than the entire CyberKnife® stereotactic radiotherapy workflow. A comprehensive institutional FMEA should include all relevant clinical personnel involved in patient care. Third, the participation of vendor‐affiliated medical physicists as facilitators may have introduced potential vendor‐related bias, although they did not independently determine the final scoring or prioritization. Fourth, because FMEA was conducted independently by site‐specific groups, the resulting RPN values should be interpreted only as relative priorities within each group and should not be directly compared across disease sites. In addition, scoring may have been influenced by institutional workflows, local clinical practice, and group composition. Therefore, the seminar‐derived failure mode lists should not be directly adopted as institutional FMEA results. This limitation has been clarified in the table captions to avoid misinterpretation. Fifth, as with other FMEA‐based studies, scoring is inherently subject to expert judgment and interobserver variability; thus, the resulting rankings should not be viewed as absolute thresholds but rather as practical tools for prioritizing discussion and guiding safety improvements.^13^

Future work should include repeated or longitudinal workshops, broader multidisciplinary participation, institution‐specific workflow mapping, postimplementation re‐evaluation of countermeasures, and integration with incident‐learning systems. Such developments would allow this educational seminar format to serve as a more effective entry point for comprehensive, institution‐specific FMEA activities and sustainable quality improvement in CyberKnife® practice.

## 5 CONCLUSIONS

This seminar allowed participants from several institutions to work through the FMEA process using actual CyberKnife® treatment workflows. Although the failure modes identified during the seminar were limited by the available time and the composition of the groups, the exercise helped participants understand how FMEA can be applied in clinical practice. The questionnaire responses also indicated that most participants gained a better understanding of risk analysis and were interested in applying it at their own institutions. The failure mode lists generated during the seminar were not intended to represent a complete FMEA for any participating institution. Rather, they may serve as practical examples for institutions beginning their own multidisciplinary risk analysis based on local workflows and clinical practices.

## AUTHOR CONTRIBUTIONS

Hiroyuki Okamoto and Shie Nishioka contributed to the study conception and design, data analysis, and manuscript preparation. Takeshi Kamomae, Kohei Okawa, Souichi Kikuchi, and Junji Suzuki contributed to seminar organization, data collection, interpretation of the results, and critical revision of the manuscript. All authors read and approved the final manuscript.

## FUNDING INFORMATION

The operation of this hands‐on seminar was supported by the Japanese Society of CyberKnife and Accuray Inc. This research was partially supported by the National Cancer Center Research and Development Fund (2025‐A‐12).

## CONFLICT OF INTEREST STATEMENT

Accuray Inc. had no role in the study design, data collection, data analysis, interpretation of the results, manuscript preparation, or the decision to submit the manuscript for publication. Hiroyuki Okamoto, Shie Nishioka, Takeshi Kamomae, Kohei Okawa, and Souichi Kikuchi received an honorarium from Accuray Inc. for serving as lecturers in this seminar. The other authors declare no competing interests.

## ETHICS STATEMENT

Ethical approval was not required for this educational seminar and questionnaire survey. Participants were informed of the purpose of the survey, that participation was voluntary, and that the collected data would be used for research and publication. Completion of the questionnaire was regarded as consent to participate.

## Supporting information



Supporting information: acm270789‐sup‐0001‐TableS1.docx

Supporting information: acm270789‐sup‐0002‐TableS2.docx

Supporting information: acm270789‐sup‐0003‐TableS3.docx

Supporting information: acm270789‐sup‐0004‐TableS4.docxs

Supporting information: acm270789‐sup‐0005‐FigureS1.tif

## Data Availability

The data that support the findings of this study are available from the corresponding author upon reasonable request.
