# Supplementary material for: A group‐based hands‐on seminar for teaching failure mode and effects analysis in CyberKnife stereotactic radiotherapy
Source: J Appl Clin Med Phys. 2026 Sep 10;27(9):e70789. doi: 10.1002/acm2.70789 (PMC13559173; doi:10.1002/acm2.70789)
Supplement: Supplementary file 1 [file ACM2-27-e70789-s002.docx]

Table S-2. Complete failure mode list for lung treatments.

| Failure Mode | *O* | *S* | *D* | *RPN* | Countermeasures^*^ |
| --- | --- | --- | --- | --- | --- |
| Irregular patient breathing | 4 | 4 | 4 | 64 | Respiratory management through coaching and visual feedback |
| Inadequate consideration of interference between the arm and the machine during the immobilization setup | 4 | 3 | 5 | 60 | Standardization of treatment positioning, collision check with the machine, and selection of the arms-down position to avoid collision |
| Contact between the machine and the patient during treatment | 4 | 3 | 5 | 60 | Establishment of an immobilization protocol, standardization of treatment positioning, checking of immobilization device size, and selection of the arms-down position to avoid collision |
| Inappropriate fiducial marker position | 4 | 4 | 3 | 48 | Information sharing with other departments and training for marker implantation |
| Fiducial marker migration | 4 | 4 | 3 | 48 | Training for marker implantation and check for marker migration (excluding symmetrical markers) |
| Difficulty maintaining the patient’s position | 3 | 4 | 3 | 36 | Enhanced patient assessment and confirmation at simulation |
| Changes in breathing pattern | 4 | 4 | 2 | 32 | Respiratory management through coaching and visual feedback |
| Changes in the correlation model | 4 | 4 | 2 | 32 | Respiratory management through coaching and visual feedback |
| Patient motion | 4 | 4 | 2 | 32 | Enhanced patient assessment, shortening of treatment time, and monitoring of patient movement by radiation technologists during treatment |
| Incorrect number of fractions | 3 | 5 | 2 | 30 | Physician peer review, physics review, and checklist (dose and fraction) |
| Incorrect instructions for immobilization | 2 | 5 | 2 | 20 | Establishment of an immobilization protocol, development of a manual, and documentation of instructions in the medical record |
| Incorrect selection of markers for tracking | 2 | 5 | 2 | 20 | Physics review, physician peer review, and standardization of the marker registration order |
| Image registration error | 2 | 5 | 2 | 20 | Physician peer review, physics review, and checklist (image registration) |
| Omission of the tracking target volume setting | 3 | 3 | 2 | 18 | Checklist (tracking target volume) |
| Error in immobilization device fabrication | 2 | 4 | 2 | 16 | Training for immobilization device fabrication and development of a manual |
| Incorrect body-surface marker placement | 2 | 2 | 3 | 12 | Physics review and recording and saving of marker positions |
| Optimization parameter error | 2 | 3 | 2 | 12 | Use of templates and training in treatment planning |
| Incorrect Monte Carlo uncertainty setting | 2 | 2 | 2 | 8 | Checklist (Monte Carlo simulation) |
| Damaged immobilization device/air leakage | 1 | 4 | 2 | 8 | Immobilization device management and pre-use check |
| Incorrect tracking target volume setting | 1 | 3 | 2 | 6 | Checklist (tracking target volume) |
| Failure to remove low-MU beams | 2 | 3 | 1 | 6 | Checklist (low-MU values) |
| Deviation in dose verification results | 1 | 5 | 1 | 5 | Reconfirmation of the verification method, recommissioning, and consideration of replanning |
| Detection of a new lesion | 4 | 1 | 1 | 4 | Physician peer review |
| Change in patient body habitus | 1 | 4 | 1 | 4 | Monitoring of general condition and pretreatment simulation |
| Incorrect selection of the tracking method | 3 | 1 | 1 | 3 | Checklist (tracking method) |
| Tracking failure (tracking volume cannot be detected because of the vertebral body, etc.) | 3 | 1 | 1 | 3 | Standardization of treatment positioning and pretreatment tracking test |

*The countermeasures were revised by the authors based on the outcomes of group discussions. FMEA: failure mode and effects analysis, *O*: occurrence, *S*: severity, *D*: detectability, *RPN*: risk priority number.
