# Supplementary material for: A group‐based hands‐on seminar for teaching failure mode and effects analysis in CyberKnife stereotactic radiotherapy
Source: J Appl Clin Med Phys. 2026 Sep 10;27(9):e70789. doi: 10.1002/acm2.70789 (PMC13559173; doi:10.1002/acm2.70789)
Supplement: Supplementary file 2 [file ACM2-27-e70789-s003.docx]

Table S-4. Complete failure mode list for prostate treatments.

| Failure Mode | *O* | *S* | *D* | *RPN* | Countermeasures^*^ |
| --- | --- | --- | --- | --- | --- |
| GTV delineation error | 4 | 5 | 4 | 80 | Physician peer review |
| Incorrect patient-specific QA evaluation criteria (potentially persistent error) | 3 | 5 | 5 | 75 | Pre-use verification during commissioning, manual development, education/training, physician review, and physics review |
| Non-optimal fiducial marker placement | 3 | 4 | 5 | 60 | Operator training and information sharing with other departments |
| Inadequate spacer placement | 3 | 4 | 5 | 60 | Operator training and information sharing with other departments |
| Incorrect CT image selection for retreatment planning | 2 | 5 | 4 | 40 | Physician peer review, physic review, and checklist (CT image selection) |
| Miscommunication of treatment planning instructions for irregular cases | 2 | 5 | 4 | 40 | Physician peer review, physic review, documentation of instructions, multidisciplinary information sharing, and plan review during conferences |
| Incorrect CT image import | 2 | 5 | 4 | 40 | Physician peer review, physic review, checklist (CT image), and plan review |
| Incorrect CT slice thickness | 3 | 4 | 3 | 36 | Physic review, verification at image import, checklist (CT image), plan review, and CT protocol standardization |
| Insufficient CT scan range | 4 | 4 | 2 | 32 | Manual development, image acquisition (or verification) by the planner, and prior information sharing with physicians regarding the treatment site and the intent/objective of the treatment plan |
| Incorrect pre-treatment patient preparation instructions (bladder filling and rectal emptying) | 2 | 3 | 5 | 30 | Review and information sharing at the morning conference on the treatment day |
| Incorrect CT selection during QA plan creation | 2 | 2 | 5 | 20 | Manual development and checklist (QA plan) |
| Inappropriate alignment center placement | 3 | 2 | 3 | 18 | Education, training, and checklist (align center) |
| Insufficient MRI image quality | 3 | 3 | 2 | 18 | Protocol development and education/training on MRI, particularly image quality |
| Air leak in the immobilization device | 2 | 3 | 2 | 12 | Pre-use verification and appropriate use of the immobilization device |
| Omission of part of the MRI sequence acquisition | 2 | 3 | 2 | 12 | Protocol organization and strict order verification |
| Incorrect immobilization device used at treatment | 1 | 3 | 3 | 9 | Pretreatment verification by the radiation therapist |
| DRR creation error | 3 | 1 | 2 | 6 | Checklist (DRR) |
| Incorrect dose calculation grid setting | 1 | 3 | 2 | 6 | Checklist (dose calculation setting) |
| Billing error | 1 | 1 | 5 | 5 | Physician peer review, physic review, and verification by the medical affair section |
| Physician order error (IMRT vs SBRT) | 2 | 1 | 2 | 4 | Physician peer review, review at the conference, and physician peer review |

*The countermeasures were revised by the authors based on the outcomes of group discussions. FMEA: failure mode and effects analysis, *O*: occurrence, *S*: severity, *D*: detectability, *RPN*: risk priority number.
