# Supplementary material for: A group‐based hands‐on seminar for teaching failure mode and effects analysis in CyberKnife stereotactic radiotherapy
Source: J Appl Clin Med Phys. 2026 Sep 10;27(9):e70789. doi: 10.1002/acm2.70789 (PMC13559173; doi:10.1002/acm2.70789)
Supplement: Supplementary file 3 [file ACM2-27-e70789-s004.docx]

Table S-1. Complete failure mode list for intracranial treatments.

| Failure Mode | *O* | *S* | *D* | *RPN* | Countermeasures^*^ |
| --- | --- | --- | --- | --- | --- |
| Contour inaccuracy | 3 | 4 | 4 | 48 | Physician peer review and checklist (small-volume contour and margin) |
| Incorrect immobilization device type | 3 | 3 | 5 | 45 | Immobilization device organization and fixation protocol |
| Incorrect prescription or fractionation instructions | 4 | 5 | 2 | 40 | Institutional protocol confirmation and information sharing for irregular cases |
| Diagnostic imaging error | 2 | 5 | 2 | 20 | Physician peer review |
| Incorrect identification of the target image location | 2 | 5 | 2 | 20 | Physician peer review, physics review, and composite evaluation with previous plans, coordination with other departments |
| Incorrect margin setting | 4 | 5 | 1 | 20 | Physician peer review, physics review, checklist (margin), and protocol development |
| Image registration error | 2 | 5 | 2 | 20 | Physician peer review, physics review, and checklist (image registration) |
| Incorrect immobilization device label | 2 | 3 | 3 | 18 | Physics review and immobilization device labeling |
| Miscommunication of immobilization instructions | 2 | 3 | 3 | 18 | Fixation protocol, immediate documentation after preparation, double-checking, and information sharing for irregular cases (documentation in the instruction sheet and morning conference) |
| Auto-contouring error | 2 | 3 | 2 | 12 | Physician peer review, physics review, checklist (contour accuracy) |
| Incorrect collimator arrangement | 2 | 2 | 2 | 8 | Checklist (Collimator selection) |
| Incorrect slice thickness | 1 | 5 | 1 | 5 | Standardization of protocol and checklist (protocol) |
| Miscommunication of the protocol | 1 | 5 | 1 | 5 | Standardized imaging orders and comments |
| Incorrect prescription or fractionation setting | 1 | 5 | 1 | 5 | Institutional protocol and checklist (dose and fraction) |
| Wrong patient | 1 | 5 | 1 | 5 | Patient identity verification |
| Failure to confirm contrast agent allergy | 1 | 5 | 1 | 5 | Enhanced pre-examination screening and electronic health record (EHR) alert display |
| Equipment interference | 1 | 5 | 1 | 5 | Demonstration and practical training |
| Incorrect immobilization device selection at treatment | 1 | 2 | 2 | 4 | checklist (immobilization) and timeout |
| Insufficient imaging range | 1 | 3 | 1 | 3 | CT acquisition protocol, scan range verification, and advance sharing with physicians regarding the treatment site and the intent (purpose) of the treatment plan |
| Incorrect dose calculation algorithm selection | 1 | 2 | 1 | 2 | Physics review and checklist (dose calculation algorithm) |
| Incorrect plan selection | 1 | 2 | 1 | 2 | Physics review, checklist (plan name), and timeout |

*The countermeasures were revised by the authors based on the outcomes of group discussions. FMEA: failure mode and effects analysis, *O*: occurrence, *S*: severity, *D*: detectability, *RPN*: risk priority number.
