# Supplementary material for: A group‐based hands‐on seminar for teaching failure mode and effects analysis in CyberKnife stereotactic radiotherapy
Source: J Appl Clin Med Phys. 2026 Sep 10;27(9):e70789. doi: 10.1002/acm2.70789 (PMC13559173; doi:10.1002/acm2.70789)
Supplement: Supplementary file 4 [file ACM2-27-e70789-s005.docx]

Table S-3. Complete failure mode list for spine treatments.

| Failure Mode | *O* | *S* | *D* | *RPN* | Countermeasures^*^ |
| --- | --- | --- | --- | --- | --- |
| Patient motion caused by pain | 3 | 3 | 4 | 36 | Enhanced patient assessment, confirmation at simulation, pain control with medication in collaboration with physicians, and motion monitoring by radiation technologists during treatment |
| Change in immobilization device shape during treatment (e.g., air leakage or deformation) | 3 | 3 | 3 | 27 | Pre-use check |
| Misregistration between MRI and CT | 2 | 4 | 3 | 24 | Physician peer review, physics review, and checklist (image registration) |
| Insufficient calculation range | 2 | 4 | 3 | 24 | Checklist (calculation range) and multidisciplinary check |
| One-vertebra misalignment during tracking | 2 | 4 | 3 | 24 | Physician review, physics review, double-check, education on image interpretation skills, and standardization of the vertebral body confirmation procedure in a manual |
| Collision between the patient and the robot | 2 | 4 | 3 | 24 | Standardization of treatment positioning and collision check with the machine |
| Collision between the arm and the machine | 2 | 3 | 3 | 18 | Standardization of treatment positioning and collision check with the machine |
| Failure to address artifacts in patients with spinal metallic implants | 2 | 3 | 3 | 18 | Physics review and standardization of the CT acquisition protocol, development of a manual for artifact management (e.g., electron density replacement and beam arrangement), and a checklist (metal artifact processing and assigned CT number) |
| Failure to set the calculation resolution to high | 2 | 3 | 3 | 18 | Checklist (dose calculation grid) |
| Residual spinal roll misalignment cannot be fully corrected | 2 | 3 | 3 | 18 | Consideration of the CTV-to-PTV margin, confirmation of imaging conditions to avoid unintended changes in contour resolution |
| Inappropriate immobilization device size: interference with the machine | 2 | 3 | 3 | 18 | Establishment of an immobilization protocol and checking of immobilization device size |
| Incorrect treatment instruction by the physician | 2 | 4 | 2 | 16 | Physician peer review, confirmation at conference |
| Incorrect MRI sequence for registration | 2 | 2 | 4 | 16 | Standardization of MR sequences, checklist (image registration), and protocol development |
| Mishandling of the patient-specific verification film (placement in the phantom) | 2 | 2 | 4 | 16 | Standardization of the verification procedure in a manual |
| Beam intersection setting error | 1 | 3 | 4 | 12 | Checklist (beam intersection) and education |
| Inappropriate immobilization device size: MR images cannot be acquired | 2 | 2 | 3 | 12 | Establishment of an immobilization protocol and checking of immobilization device size |
| Incorrect CT scan range: tracking cannot be performed | 2 | 2 | 3 | 12 | Standardization of the CT acquisition protocol, advance sharing with physicians regarding the treatment site and the intent/purpose of the treatment plan |
| Inappropriate alignment center placement | 2 | 2 | 3 | 12 | Education, training, and checklist (align center) |
| Incorrect chamber measurement point during patient-specific verification | 2 | 2 | 3 | 12 | Physics review and standardization of the verification procedure in a manual, and education |
| Wrong treatment site (incorrect vertebral level) | 1 | 5 | 2 | 10 | Physician peer review, physics review, collaboration with other departments, and advance sharing with physicians regarding the treatment site and the intent/purpose of the treatment plan |
| Wrong patient | 1 | 5 | 2 | 10 | Barcode verification, etc. |
| Incorrect selection of the conversion table | 1 | 3 | 3 | 9 | Checklist (CT number-to-electron density conversion table) |
| Misidentification of the approved plan | 1 | 3 | 3 | 9 | Standardization of the operating procedure in a manual, cautionary alert, and naming rules |
| Incorrect approved plan selected when two plans exist for the same site: inappropriate plan selected by mistake | 1 | 3 | 3 | 9 | Physics review, checklist (plan approval), and naming rules |
| Use of demo mode on a patient: unnecessary irradiation | 1 | 3 | 3 | 9 | Physics review and restriction on the use of demo plans |
| Incorrect number of fractions | 1 | 4 | 2 | 8 | Checklist (dose and fraction) |
| Inclusion of full-width characters (e.g., Japanese characters) in external image data | 1 | 2 | 4 | 8 | Pretransfer confirmation and standardization and manual preparation |
| Emergency patient buzzer in the treatment room does not sound because of battery depletion | 1 | 2 | 4 | 8 | Pre-use check |
| Accidental deletion of the correct plan | 1 | 2 | 4 | 8 | Standardization of the operating procedure in a manual, cautionary alert, and naming rules |
| Error in CT acquisition rules (e.g., cervical spine irradiation should use skull tracking) | 1 | 2 | 3 | 6 | Physics review and standardization of the CT acquisition protocol |
| Incorrect CT acquisition parameters (e.g., tube voltage) | 1 | 3 | 1 | 3 | Physics review and establishment of the CT acquisition protocol |

*The countermeasures were revised by the authors based on the outcomes of group discussions. FMEA: failure mode and effects analysis, *O*: occurrence, *S*: severity, *D*: detectability, *RPN*: risk priority number.
